# A Case of COVID-19 Pneumonia in a Young Male with Full Body Rash as a Presenting Symptom

**DOI:** 10.5811/cpcem.2020.3.47349

**Published:** 2020-03-28

**Authors:** Madison Hunt, Christian Koziatek

**Affiliations:** New York University School of Medicine, Department of Emergency Medicine, New York, New YorkBellevue Hospital Center, Department of Emergency Medicine, New York, New York

**Keywords:** COVID-19, coronavirus, rash

## Abstract

**Background:**

In December 2019 the coronavirus disease of 2019 (COVID-19), caused by the severe acute respiratory syndrome coronavirus 2, was identified in Wuhan, China. In the ensuing months, the COVID-19 pandemic has spread globally and case load is exponentially increasing across the United States. Emergency departments have adopted screening and triage procedures to identify potential cases and isolate them during evaluation.

**Case Presentation:**

We describe a case of COVID-19 pneumonia requiring hospitalization that presented with fever and extensive rash as the primary presenting symptoms. Rash has only been rarely reported in COVID-19 patients, and has not been previously described.

## CASE PRESENTATION

**Image 1:**
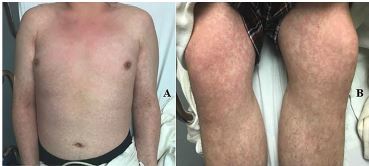
Image of the anterior trunk and upper extremities (A) and anterior lower extremities (B) demonstrating a diffuse, morbilliform, maculopapular rash.

**Image 2:**
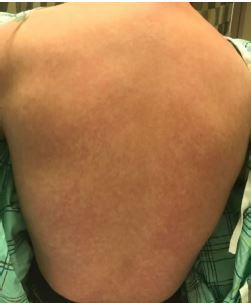
Image of the posterior trunk similarly demonstrates a diffuse, maculopapular morbilliform rash.

**Image 3:**
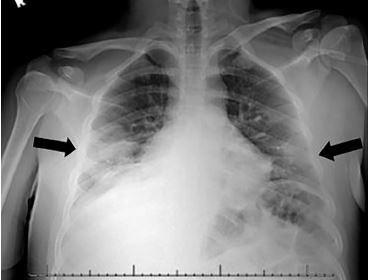
A chest radiograph demonstrating bilateral infiltrates consistent with bilateral multifocal pneumonia.

A 20-year-old previously healthy male originally presented to an urgent care center with a chief complaint of fever and rash. He was diagnosed with a viral upper respiratory infection and sent home with supportive care. Six days later, the patient presented to the emergency department (ED) with continued fever and rash. Vital signs included a temperature of 103.0˚ Fahrenheit, heart rate 115 beats per minute, blood pressure 93/54 millimeters of mercury, respiratory rate 24 breaths per minute, and an oxygen saturation of 91%. Physical examination revealed a diffuse, morbilliform rash across the trunk and extremities, sparing the face (Images 1 and 2). There was no mucosal or ocular involvement. Chest radiograph revealed bilateral infiltrates consistent with multifocal pneumonia (Image 3). Labs included a normal leukocyte count (8300 units per liter [uL], reference range 4200–9100/uL) with an absolute lymphocyte count of 800/uL (reference range 1300–3600/uL). A C-reactive protein was elevated at 118.5 milligrams per liter (mg/L) (reference range 0–5 mg/L). A rapid strep test and an human immunodeficiency virus test were both negative, as was a respiratory viral panel. The patient required escalating amounts of supplemental oxygen during his ED course and was admitted to the intensive care unit (ICU). A severe acute respiratory syndrome coronavirus 2 (SARS-CoV-2) polymerase chain reaction test resulted positive on hospital day two. The patient remains hospitalized in the ICU on hospital day six.

**CPC-EM Capsule**

What do we already know about this clinical entity?

Coronavirus disease of 2019 (COVID-19) typically initially presents with symptoms similar to other viral respiratory infections, most commonly with fever, cough, fatigue, myalgias, and congestion.

What is the major impact of the image(s)?

This case describes a COVID-19 patient who presented with a full body rash, which is a rare presenting symptom in previous studies and has not been described previously in the literature.

How might this improve emergency medicine practice?

COVID-19 may rarely present with an associated morbilliform viral eruption and should not be discarded as a diagnostic possibility in patients with viral syndrome and rash.

## DISCUSSION

We describe a case of COVID-19 pneumonia in a young, healthy male requiring hospitalization, which presented with fever and extensive rash. The rash was morbilliform, maculopapular, and nonpruritic, and appeared consistent with a viral exanthem. The clinical characteristics of COVID-19 have been described in several publications, most thoroughly in a case series of 1099 patients by Guan et al. Fever, cough, congestion, and dyspnea are the most common presenting symptoms. Only 2/1099 patients were noted to have any skin rash, and the rash was not described^1^; no other publications have noted or described skin manifestations as a presenting symptom.^1^–^5^ Rash may be a rare presenting symptom of COVID-19 and should be kept in mind by front-line providers.
